# Visual Outcomes in Ectopia Lentis in Marfan Syndrome: A Study of Four Surgical Techniques in Children and Adults

**DOI:** 10.3390/medicina60071098

**Published:** 2024-07-05

**Authors:** Anca Cristina Dogaroiu, Maria Dudau, Catalin Dogaroiu, Calin Petru Tataru

**Affiliations:** 1 Alcor Clinic, 030829 Bucharest, Romania; cristina.dogaroiu@drd.umfcd.ro (A.C.D.); calin.tataru@umfcd.ro (C.P.T.); 2 Laboratory of Biochemistry, “Victor Babes” National Institute of Pathology, 050096 Bucharest, Romania; dudau_maria2002@yahoo.com; 3 Department of Cell Biology and Histology, “Carol Davila” University of Medicine and Pharmacy, 020021 Bucharest, Romania; 4 Department of Morphological Sciences, “Carol Davila” University of Medicine and Pharmacy, 020021 Bucharest, Romania; 5 Department of Ophthalmology, “Carol Davila” University of Medicine and Pharmacy, 020021 Bucharest, Romania; 6 Clinical Hospital for Ophthalmological Emergencies, 010464 Bucharest, Romania

**Keywords:** Marfan syndrome, ectopia lentis surgical techniques, modified Cionni capsular tension rings

## Abstract

*Background/Objectives*: To evaluate how the surgical technique and type of implanted intraocular lens influence the postoperative visual acuity and complications in ectopia lentis associated to Marfan syndrome patients. *Materials and Methods*: The medical records and videos of ectopia lentis surgeries in patients (children and adults) with Marfan syndrome, were retrospectively reviewed and compared. The study included 33 eyes that underwent four different intraocular lens implantation (IOL) techniques: IOL in conjunction with a simple capsular tension ring, IOL in conjunction with a Cionni modified capsular tension ring (m-CTR), two-point scleral IOL fixation and IOL with one haptic in the bag and one haptic sutured to the sclera. *Results*: Vision significantly improved from a mean preoperative visual acuity of 0.1122 to a mean postoperative visual acuity of 0.4539 in both age groups (*p* < 0.0001), with no difference in the primary outcome between children and adults. The most common surgical technique used in both age groups was IOL in conjunction with an m-CTR. There was only one major postoperative complication requiring additional surgery. *Conclusions*: Zonular weakness mainly influenced by age was the most important selection criterion for the surgical approach. Regardless of the technique employed, the postoperative visual acuity was improved in both adults and children.

## 1. Introduction

Marfan syndrome is an autosomal dominant inherited disorder that affects the connective tissue. It is caused by mutations in the fibrillin-1 gene (FBN1), which modifies fibrillin production [1]. There have been identified over 3000 possible mutations of the FBN1 gene [2]. Because fibrillin is the major component of the extracellular matrix [3], its abnormal production affects numerous organs and systems; the most frequent being the cardiovascular, skeletal and ocular system. Marfan syndrome diagnosis is based on the revised Ghent nosology from 2010 [4]. The only major diagnostic criterion for the ocular system is the presence of ectopia lentis [4], which is the displacement of the crystalline lens from its anatomical position. In Marfan syndrome this is the most frequent manifestation involving the ocular system [5]. In general, lens displacement affects both eyes with varying degrees of luxation [6]. Patients with significant ectopia lentis complain of fluctuating or blurry vision or monocular diplopia. For these cases the only treatment is surgical removal of the natural crystalline lens, followed by an artificial intraocular lens (IOL) implant.

The aim of this retrospective comparative study is to evaluate four surgical techniques used in ectopia lentis in children and adults with Marfan syndrome and postoperative results in terms of visual acuity and complications.

## 2. Materials and Methods

This study was conducted in the Clinical Hospital for Ophthalmological Emergencies, Bucharest, Romania, according to the Declaration of Helsinki, being approved by the Ethics Committee of the hospital. The study was based on reviewing medical records and videos of ectopia lentis surgeries in patients (children and adults) with Marfan syndrome performed by the same surgeon between February 2012 and June 2020. Informed consent for patient information and images to be published was provided by the patients or their authorized representatives.

Four methods of IOL implantation were performed and evaluated in this study: IOL implantation in the bag along with a simple capsular tension ring (CTR) (group 1), IOL implantation in the bag along with a scleral fixated Cionni CTR (group 2), scleral IOL fixation with suture in two points (group 3) and IOL implantation with one haptic in the bag and one haptic sutured to the sclera (group 4).

The age, gender and full ophthalmic exams of patients included in the study were retrieved and analysed. The ophthalmic exams included: preoperative and postoperative best-corrected visual acuity (BCVA) using a Snellen chart, slit lamp examination of anterior segment and eye fundus, intraocular pressure (IOP) measurements by a non-contact tonometer, Topcon CT-80 NCT (Topcon Medical Systems, Paramus, NJ, USA) in cooperative children and adults or under sevofluorane sedation with Tono-Pen XL (Reichert Inc., Depew, NY, USA) in incompliant children and B-scan ultrasonography in those cases where the fundus of the eye could not be examined due to a completely opaque crystalline lens. For cooperative patients, biometry was performed using an optical biometer (IOL Master500, Carl Zeiss Meditec, Jena, Germany), while for non-cooperative patients, contact ultrasound biometry was performed (Ultrascan Digital 2000 contact ultrasound A-scan, Alcon, Geneva, Switzerland), along with keratometric measurements taken with an autorefractokeratometer (Retinomax, Righton, Tokyo, Japan). The Sanders–Retzlaff-Kvaff/T (SRK/T) and Holliday–Segar formulas were applied to calculate the IOL power. We used optimized constants: for three-piece lenses, constant 118.4, and for single-piece lenses, constant 118.7. The postoperative refraction target was emmetropia or up to 1D hyperopia for children.

Surgical treatment was pursued in severe lens subluxation when the lens equator was in the visual axis and also in patients with low visual acuity after best corrected visual acuity under 0.014 Snellen. Patients with crystalline lens removal followed by IOL implantation in the same surgery session were included in the study. Exclusion criteria: patients with advanced glaucoma, ocular inflammation, retinal diseases, keratoconus and pterygium.

Postoperatively, the patients were examined after 1 day, 1 week, 1 month and 6 months. The BCVAs of the last follow-up were selected. Visual acuity was measured using the Snellen value, which was used to show the distribution of preoperative and postoperative BCVA. Complications were recorded after thorough ophthalmic examinations.

### 2.1. Statistical Analysis

For the descriptive purpose of our study, quantitative variables were expressed as means and standard deviations. Numerical variables before and after surgery were compared using student’s *t* test for paired and unpaired samples. For each technique we assessed the following parameters: mean patients’ age, preoperative BCVA (preop BCVA), postoperative BCVA (postop BCVA), axial length and astigmatism. The data were analyzed by Wilcoxon test and Mann–Whitney test. All statistical analyses were performed using IBM SPSS Statistics v. 25. Statistical significance was defined as a *p* < 0.05 (two-tailed test).

### 2.2. Surgical Technique Descriptions

Each adult case was carried out under local anesthesia and general anesthesia in children, followed by proper skin preparation using povidone-iodine and surgical draping.

**Figure 1:**
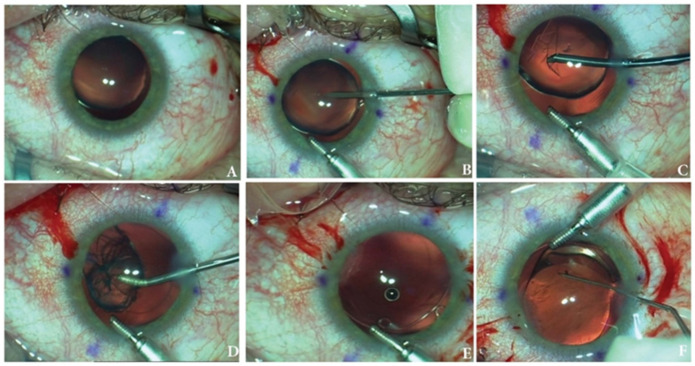
Surgical technique of IOL implantation in the bag combined with a simple capsular tension ring. (**A**) Initial intraoperative aspect of the lens; (**B**) puncturing the anterior capsule; (**C**) capsulorhexis; (**D**) crystalline lens manual aspiration; (**E**) aspect of the capsular bag after CTR implantation; (**F**) IOL and CTR in the capsular bag at the end of the surgery.

First type of surgical technique: IOL implantation in the bag combined with a simple capsular tension ring (Figure 1).

Technique description. Three 1.2 mm incisions were performed in the cornea as follows: one for the anterior chamber maintainer, another one for the side port and the last one for the main incision. Afterwards, the main incision was enlarged to 2.2 mm and capsulorhexis was created using a microforceps. Depending on its hardness the crystalline lens was removed by either manual aspiration or phacoemulsification. After thorough polishing of the capsular bag, a simple capsular tension ring was implanted in the bag under protection of ophthalmic viscoelastic devices (OVDs). In the bag, the IOL was implanted horizontally or, when using toric IOL, on the proper axis. Surgery ended with careful aspiration of OVDs and stromal hydration of the incisions.

**Figure 2:**
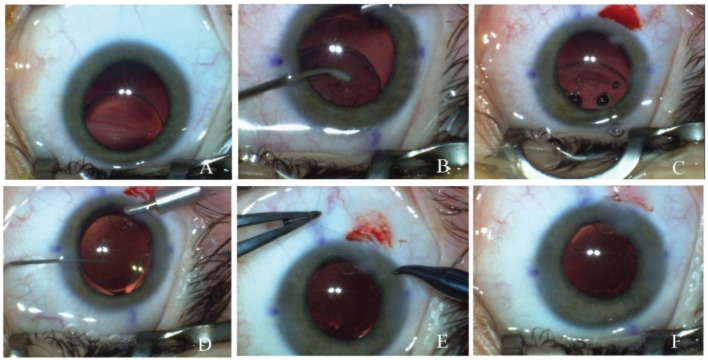
Surgical technique of IOL implantation in the bag combined with a scleral fixated Cionni CTR. (**A**) Initial intraoperative aspect of the lens; (**B**) aspiration of the crystalline lens material; (**C**) scleral pocket performed in the area of maximum subluxation; (**D**) insertion of the Cionni CTR and IOL in the capsular bag; (**E**) centering the IOL and the Cionni CTR by knotting the two threads; (**F**) view at the end of the surgery.

Second type of surgical technique: IOL implantation in the bag combined with a scleral fixated Cionni CTR (Figure 2).

Technique description. The area of maximum lens subluxation was marked with a sterile pencil. Incisions, capsulorhexis and lens material removal were performed as described in the first technique. Due to the higher degree of subluxation, a capsular bag retractor may be necessary to temporarily steady the bag. In the area of maximum subluxation, a conjunctival peritomy was performed. In the same area, starting from the limbus, a scleral pocket was made using a crescent knife. At 2 mm from the limbus, around the scleral flap, the straight needle of a polypropylene 10.0 suture was introduced in the eye. The needle passed in the eye and exited through the main incision. Afterwards, the needle of the polypropylene 10.0 suture was placed through the fixation hook of a modified Cionni capsular tension ring. Under OVDs, the Cionni ring was implanted in the eye and manipulated so that the fixated hook was placed around the area of maximum subluxation. The straight needle of the 10.0 polypropylene suture was introduced through the main incision and with the help of a bent 30 G needle was externalized at 1.5 mm lateral to the first suture. The artificial lens was implanted in the bag and the OVDs were thoroughly washed out. The two polypropylene sutures were brought out of the scleral pocket and trimmed. The bag–Cionni CTR–artificial lens complex was centered by knotting the two threads. Afterwards, the two sutures were trimmed again and buried into the scleral pocket. Corneal incisions were sealed by stromal hydration and a conjunctival suture was performed if needed.

**Figure 3:**
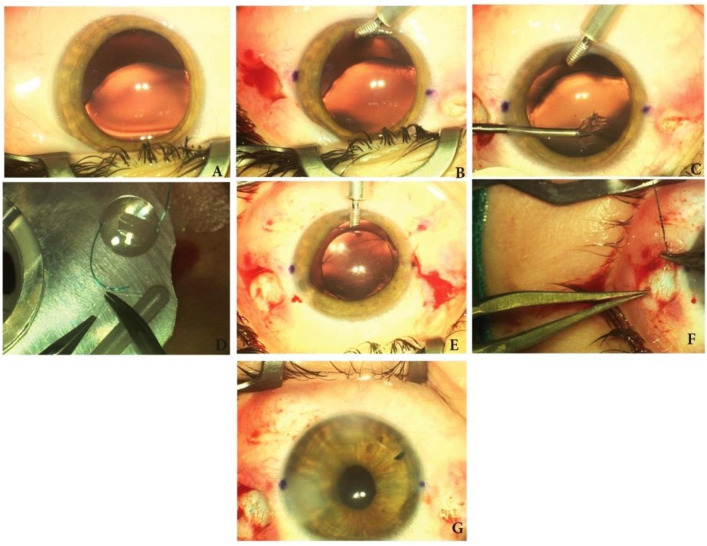
Surgical technique of scleral IOL fixation with suture in two points. (**A**) Intraoperative aspect of the lens; (**B**) two diametrically opposed conjunctival peritomies were performed; (**C**) capsulorhexis; (**D**) tying the suture to the IOL haptic; (**E**) aspect after IOL implantation; (**F**) anchoring the IOL haptics to the sclera; (**G**) end of the surgery before placing conjunctival sutures.

Third type of surgical technique: scleral IOL fixation with suture in two points (Figure 3).

Technique description. Three incisions were performed and an anterior chamber maintainer was mounted. Due to the zonular laxity, the capsulorhexis was initiated with a needle and continued with a microforceps. After the capsulorhexis was performed, the crystalline lens was separated from the capsular bag through hydrodissection. The crystalline lens material was removed by manual aspiration or by phacoemulsification. At this stage, the capsular bag could be left in place or could be removed if it was damaged. An anterior vitrectomy was performed. On the limbus, two opposed horizontal points were marked. Adjacent to these points at 2.5 mm from limbus, a conjunctival peritomy was performed. The main incision was enlarged to 3 mm and OVDs were injected in the eye. A straight needle of a 10.0 polypropylene double-armed suture was passed in the eye at 2 mm from the limbus. The straight needle exited the eye through the main incision using a 30 G bent guiding needle. Opposite to the first suture, the straight needle of a second 10.0 polypropylene double-armed suture was inserted at 2 mm from the limbus. In order to exit through the main incision, this suture needs to be first externalized through the side port and afterwards passed through the main incision. The two straight needles were cut. The end of the first curved needle located on the 3 o’clock meridian was knotted at 1.5 mm, on the first haptic of a three-piece intraocular lens. The end of the second curved needle located on the 9 o’clock meridian was knotted on the second haptic of the three-piece intraocular lens. The intraocular lens was then folded using two forceps and implanted into the eye. Afterwards it was centered by simultaneously pulling the two opposed sutures. OVDs were washed out and the incisions were hydrated. To stabilize the suture on each side, the curved needle was passed four or five times through the sclera in a serpentine manner. The sutures were cut near to the sclera and covered up with conjunctiva. The surgery ended by suturing the conjunctiva.

Fourth type of surgical technique: IOL implantation with one haptic in the bag and one haptic sutured to the sclera.

Technique description. Three corneal incisions were performed and an anterior chamber maintainer was fixed. The main incision was enlarged to 2.2 mm. Using a microforceps, the capsulorhexis was performed under continuous infusion of balanced salt solution (BSS). Lens material was separated from the capsular bag by hydrodissection and it was removed using manual aspiration or phacoemulsification. The BSS infusion was turned off and OVDs were injected into the eye. At 2.5 mm from the limbus, a conjunctival peritomy was made around the maximum point of subluxation. The straight needle of a 10.0 polypropylene double armed suture was inserted in the eye, at 2 mm from the limbus, in the area of conjunctival peritomy. After the straight needle exited the eye through the main incision, it was passed through the intraocular lens cartridge. The suture attached to the straight needle was cut, leaving only 10 cm of suture attached to the curved needle. The end of the suture was knotted to the distal portion of a three-piece intraocular lens haptic. After the intraocular lens was folded and placed into the cartridge, it was injected in the anterior chamber. From this position, the haptic with the suture was placed in the sulcus and the free haptic in the capsular bag. OVDs were washed out and the incisions were hydrated. The curved needle was passed 4–5 times through the sclera in a serpentine manner. The conjunctiva was sutured with separate threads.

## 3. Results

The study lot consisted of 33 eyes (19 patients), including 16 female eyes (48.5%) and 17 male eyes (51.5%), with a mean age at the time of surgery of 17.39 ± 11.75 years, ranging from 4 to 42 years, and 48.5% of the patients being children (under 18 years of age) and 51.5% being adults (above 18 years of age). Of the 33 eyes, 16 were right eyes and 17 left eyes. Based on the surgical technique performed on each eye, the study lot was divided as follows: group 1—IOL implantation in the bag combined with a simple capsular tension ring—which was performed on 4 eyes, group 2—IOL implantation in the bag combined with a scleral fixated m-CTR—on 22 eyes, group 3—scleral IOL fixation with suture in 2 points—on 5 eyes and group 4—IOL implantation with one haptic in the bag and one haptic sutured to the sclera—on 2 eyes, with children/adults distribution for each group shown in Table 1. Sample size will be estimated as follows: assuming the incidence of complications in patients with ectopia lentis is 15% and the margin of error is 20%. For a 5% significance level, Z_α/2_ is 1.96 for the two-tailed alternative hypothesis with a sample size of 27.

For each technique we assessed the following parameters (Table 2): mean patients’ age, preoperative BCVA (preop BCVA), postoperative BCVA (postop BCVA), axial length and astigmatism. The data were analyzed by Mann–Whitney test regarding pre- and postoperative BCVA.

Postoperative BCVA improved significantly in all 33 eyes (*p* < 0.05) but we obtained a high statistical significance in group 2 technique when compared with the preoperative BCVA (*p* < 0.05). Significant visual acuity improvement was observed in eyes with IOL implantation in the bag combined with a scleral-fixated Cionni m-CTR, but there was no statistical difference between techniques; all techniques improved BCVA (Table 2). Two cases were lost within the 6-month follow-up period. Regarding BCVA distribution between these four techniques, the lowest BCVA of 0.1 was observed in a patient with scleral IOL fixation with sutures in two points. The highest BCVA was seen in a patient using the same surgical technique. In the study lot, 18 eyes had a BCVA between ≥0.3 and <0.8, which is considered a moderate visual acuity loss according to the International Council of Ophthalmology [7], with the normal BCVA being ≥0.8.

For statistical analysis, due to small groups, we compared technique 2 (IOL implantation in the bag combined with a scleral fixated Cionni CTR) with the other three techniques (group 1—IOL implantation in the bag combined with a simple capsular tension ring—which was performed, group 3—scleral IOL fixation with suture in 2 points and group 4—IOL implantation with one haptic in the bag and one haptic sutured to the sclera) to rule out differences in the visual acuity BCVA preoperative and postoperative (logMAR) (Table 3). Mann–Whitney and Kruskal–Wallis tests was used between pre- and postoperative BCVA (logMAR) in each group and between technique 2 and the 3 others. No differences were observed in age, astigmatism or IOP between the main technique and the other grouped techniques, except for variations in the IOL type due to specific characteristics described in the Section 2.

**Figure 4:**
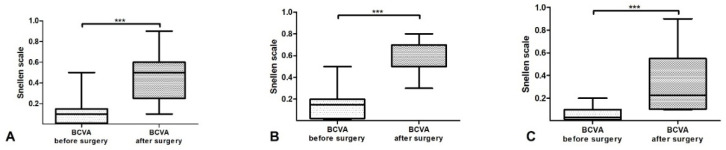
BCVA diagram before and after surgery (*** *p* value): (**A**) whole study lot (*p* = 0.001); (**B**) adults (*p* = 0.001); (**C**) children (*p* = 0.012) (Wilcoxon Signed Ranked Test and ANOVA).

The mean preoperative BCVA and postoperative BCVA was 0.1122 and 0.4539, respectively (SD ± 0.1114–0.248). The minimum preoperative BCVA was 0.014 and minimum postoperative BCVA was 0.1. The maximum preoperative BCVA was 0.5, while the maximum postoperative BCVA was 0.9 (Figure 4A). We also analyzed postoperative BCVA improvement, separately for children and adults. In children (Figure 4B), we obtained *p* < 0.0003, with a minimum preoperative BCVA of 0.014 and a minimum postoperative BCVA of 0.1. The maximum preoperative BCVA was 0.2 and postoperative BCVA was 0.9 (mean preoperative BCVA was 0.0765 and mean postoperative BCVA was 0.3247 with SD ± 0.05813–0.2173). In adults (Figure 4C), we obtained *p* < 0.0007 with a minimum preoperative BCVA of 0.014 and a minimum postoperative BCVA of 0.3. The maximum preoperative BCVA was 0.5 and the maximum postoperative BCVA 0.8 (mean preoperative BCVA was 0.1459 and mean postoperative BCVA was 0.5750 with SD ± 0.1384–0.1571). Postoperative BCVA for the entire study group showed a statistically significant improvement (*p* < 0.0001), despite two missing patients from the follow-up measurements.

For 32 eyes (for one eye the AL and keratometric data were unavailable), we analyzed the value of the flattest meridian (K1), the value of the steepest meridian (K2), as well as the average of the two meridians (Kmed). We obtained a minimum K1 of 35.68 D, a maximum K1 of 45.24 D and an average of 40.72 ± 2.37 D. For K2, we obtained a minimum of 38.66 D, a maximum of 50.29 D and an average of 42.86 ± 2.77 D. The Kmed values had a minimum of 37.17 D, a maximum of 47.27 D and an average of 41.83 ± 2.46 D. The keratometric values were roughly homogeneous throughout the study lot; the mean values of these measurements are presented in Table 4.

The type of astigmatism was separately evaluated for children and adults, according to the location of the steepest meridian (Table 5).

The descriptive statistical analysis of the axial length (AL) for males and females is shown in Table 6. There was no difference between males and females in relation to axial length (*p* = 0.440, 24.13 ± 2.06 mm in males and 23.46 ±1.05 mm in females). The confidence interval level was set to 95% where a corresponding *p* value threshold was identified as 0.05.

The AL values for children and adults were grouped in three categories, <22 mm, 22–25 mm and >25 mm [8], to analyze whether there are differences in distribution based on age in ectopia lentis. The values for age and axial length were analyzed by the Mann–Whitney U test and the differences between groups were not statistically significant (*p* = 0.213). These results are presented in Table 7.

**Figure 5:**
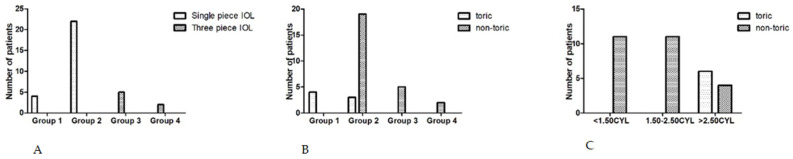
IOL type implanted taking into account technique and astigmatism: (**A**) distribution of single-piece IOL and three-piece IOL in each technique group; (**B**) distribution of toric and non-toric IOL in each technique group; (**C**) correlation between value of the astigmatism and the type of implanted IOL.

The single piece IOL design was implanted in group 1 and 2 surgical technique cases, representing 78.8% from the study lot and the three piece IOL was implanted in group 3 and 4, representing 21.2% (Figure 5A). For the correction of astigmatism, toric IOLs were used in group 1 and in small number of cases (3 out of 22) in group 2 (when the bag was suitable for proper IOL placement and centration) and in all other cases from this group, non-toric IOLs were used. In the surgical technique group 3 and 4, only non-toric IOLs were implanted (Figure 5B). We considered three categories of preoperative astigmatism: <1.50 Cyl, 1.50–2.50 Cyl and >2.50 Cyl. In the first two categories we only implanted non-toric IOLs. In the third category of preoperative cylinder values >2.50 Cyl, the toric IOL was implanted in six eyes and a non-toric IOL in four eyes (Figure 5C).

In our study, there were two postoperative complications, one of which was major, consisting of the untying of the 10.0 polypropylene suture, leading to notable decentering of the Cionni ring–capsular bag–IOL complex. This required immediate surgical reintervention to replace the suture. The other complication was a slight decentration of the lens without an impact on visual acuity or intraocular pressure.

None of the eyes presented pupillary abnormalities due to surgical interventions, except for those associated with Marfan syndrome.

## 4. Discussion

Ectopia lentis is the most frequent ocular finding in Marfan syndrome and its surgical correction is considered a challenge due to anatomical and structural alterations of the zonular fibers. Therefore, in order to achieve optimal postoperative results, various surgical approaches were designed and implemented. Usually, a surgeon performs one or two techniques, based on experience, IOL type available and postoperative results; hence most of the studies present postoperative results from up to two surgical techniques [9,10]. To our knowledge, this is the only study that assesses four surgical techniques employed by the same surgeon to treat ectopia lentis in patients with Marfan syndrome.

The gender distribution of patients in the study group showed no significant differences between females and males, with our results being similar to those reported by other authors [11,12,13].

A patient’s age is an important factor in the recommendation to undergo surgery [14], selection of the surgical approach and visual prognosis. The mean age of our study group was 17.39 ± 11.75 years, which is not highly different from that reported by Sinha et al., 19.80 years old [15]. However, the minimum age of patients in our study was 4 years old, whereas in Sinha’s study it was 16 years old [15]. A younger age at the time of surgery is associated with a higher degree of subluxation [16]; thus, surgery is recommended as soon as possible due to poor visual acuity. Other factors that influence the timing of surgery are the risk of amblyopia [17] and uncertain visual prognosis. The study of Erdogan [18] based on a similar number of eyes (35) with a mean age of 12 ± 7.14 and a minimum age of 5 years old, presented three different techniques associated with IOL implantation in the same session.

Postoperative results showed a significant improvement of the visual acuity for both children and adults. A thing to remember is that in pediatric patients, visual acuity can be further augmented by amblyopia treatment. Therefore, parents should be warned that the surgery is just the first step in the visual recovery of their children.

Our keratometric measurements showed a flat cornea, in accordance with results of previous studies [19,20,21]. Axial length for children and adults had similar results for all three axial length groups, revealing no correlation between age and axial length. A similar conclusion was obtained by Chen in his large study [22].

With-the-rule astigmatism was the most frequent type in both children and adults, followed by oblique astigmatism; findings also reported by Chen [22]. None of our patients had against-the-rule astigmatism, which was found by Chen in 11.2% of his cases [22]. A possible explanation could be attributed to the large number of eyes (215 eyes) enrolled in Chen’s study compared to ours.

In Marfan syndrome, ectopia lentis is caused by fibrillin abnormalities [17], which induce an abnormal lens movement (phacodonesis). In ectopia lentis surgery, phacodonesis and the lack of zonular counter tension make it more difficult to perform capsulorhexis, aspiration and phacoemulsification. Furthermore, in children and young adults the capsular bag is more elastic, which makes capsulorhexis even more difficult [23]. Among the four types of surgical techniques assessed in our study, the one that implies suturing the intraocular lens to the sclera was more frequent in children (four children and one adult), because younger ages associate with a higher degree of lens subluxation, which impedes capsular bag preservation [15]. On the other hand, the technique that entails bag stabilization using a simple capsular tension ring was only used in adults (four cases), justified by the lower degree of lens subluxation in this age group; this provided a good visual acuity, which postpones the surgery. In our study, the most frequent technique involved the suture of a modified capsular tension ring to the sclera, followed by intraocular lens implantation. Although this represents a surgical challenge even in the hands of an experienced surgeon, we considered it the best approach in ectopia lentis surgery. Chen also believed that using a modified capsular tension ring was the best option in ectopia lentis surgery [13]. In the study of Erdogan et al., three different techniques were compared (intrascleral fixation of IOL, scleral IOL fixation with the Z-suture and IOL implantation with the use of a Cionni m-CTR), with no significant differences in terms of postoperative results and complications [18]. The preferred surgical approach of Halpert in young children with ectopia lentis was pars plana or limbal lensectomy [23]. The resulting aphakia was corrected afterwards by contact lenses or eyeglasses. These methods for correcting aphakia in children have many disadvantages [24]: eyeglasses—poor vision, narrowed visual field, hard to wear and contact lenses—expensive, hard to wear, risk of irritation and infections. Rezar-Dreindl et al. analyzed the visual outcome in children with ectopia lentis in Marfan syndrome after lensectomy (with/without IOL implantation) or conservative treatment, showing an improvement in both methods (83% vs. 75%) [25]. Other approaches cited in the literature are artisan lens implantation, iris sutured IOL and sutureless scleral fixation, but none of these are considered as the gold standard [26,27,28].

Postoperative visual acuity is increased by correcting preoperative astigmatism with toric intraocular lenses. In our study, in 6 out of 10 cases where preoperative cylinder was higher than 2.50 D we successfully implanted a toric intraocular lens. The surgical technique imposed both the type (toric or non-toric) and design of the intraocular lens implanted. Toric intraocular lenses were only implanted in those cases where the capsular bag was stabilized by simple or modified capsular tension rings.

One important concern was the preservation of the capsular bag, which was accomplished in 28 eyes (out of the total of 33 eyes). In these 28 eyes, we implanted a single- piece intraocular lens in 26 eyes and a three-piece intraocular lens in 2 eyes. In 5 eyes the bag could not be preserved due to a high degree of subluxation. In these cases, we sutured a three-piece intraocular lens to the sclera. A single-piece intraocular lens could also be sutured to the sclera, but the design of the three-piece is more appropriate for this technique.

In our study there were two postoperative complications. The most serious was the untying of the 10.0 polypropylene suture resulting in a significant decentration of the Cionni ring—capsular bag—IOL complex, imposing surgical reintervention to replace the suture. The other complication was a slight decentration of the lens without impact on visual acuity or intraocular pressure.

The drawbacks of the study were the short follow-up time and the relatively small number and uneven distribution of patients in the four groups of techniques.

## 5. Conclusions

The surgical correction of ectopia lentis is considered a challenge due to anatomical and structural alterations of the zonular fibers. Our study evaluated four different surgical techniques for ectopia lentis in children and adults, in terms of postoperative visual acuity and complications. The most frequent technique, which in our opinion best reconstructs the zonular anatomy, involved the suture of a modified capsular tension ring to the sclera, followed by in-the-bag intraocular lens implantation. In adults with a mild degree of lens subluxation, bag stabilization was possible only using a simple capsular tension ring. Suturing the intraocular lens to the sclera was performed in those cases with severe lens subluxation, mostly in children. The least applied technique was the IOL implantation with one haptic in the bag and one haptic sutured to the sclera.

Regardless of the technique employed the postoperative visual acuity was improved in both adults and children.

## Figures and Tables

**Table 1:** Number of eyes (children and adults) for each surgical technique.

	Group 1 (n = 4)	Group 2 (n = 22)	Group 3 (n = 5)	Group 4 (n = 2)
Adults (n = 17)	4	11	1	1
Children (n = 16)	0	11	4	1

**Table 2:** Descriptive statistical analysis between techniques * Mann–Whitney test).

	Group 1 (n = 4)	Group 2 (n = 22)	Group 3 (n = 5)	Group 4 (n = 2)
Age (years)	24 ± 6	17.90 ± 12.69	12 ± 10.17	12 ± 11.31
Preop BCVA	0.183 ± 0.1549	0.122 ± 0.11	0.0348 ± 0.03727	0.057 ± 0.0608
Postop BCVA	0.55 ± 0.173 0.068 (*)	0.441 ± 0.2 0.0001 (*)	0.44 ± 0.357 0.42 (*)	0.453 ± 0.22 0.317 (*)
Axial length (mm)	24.60 ± 2.78	23.55 ± 1.06	24.59 ± 2.479	22.75 ± 2.1
Astigmatism	1 ± 1	1.3 ± 0.79	1.18 ± 0.59	3.75 (one value)

**Table 3:** Statistical analysis between techniques with mean descriptive statistics (Mann–Whitney **, Kruskal–Wallis *).

	Tehnique 2 (22 Eyes)	Tehnique 1, 3, 4 Mean (11 Eyes)	*p* Value *
Age	17.9091 ± 12.69762	16.3636 ± 10.08239	0.818
Axial length	23.5529 ± 1.06927	24.2664 ± 2.40488	0.751
BCVA preop	0.9173 ± 0.25051	0.9473 ± 0.31953	0.643
BCVA postop	0.4105 ± 0.22319 ** 0.0001	0.4130 ± 0.33320 ** 0.005	0.798
IOL	Single piece in all cases	Three piece in 7 cases and single piece in 4 cases	
Complications	1 major	1 minor	
IOP	17.3182 ± 1.32328	17.7 ± 4.13790	0.567
K mean	41.9648 ± 2.20591	41.5991 ± 3.01131	0.678

**Table 4:** The mean values of keratometry.

	Mean
Keratometry (Kmed)	41.83 D ± 2.46 D
K1	40.72 D ± 2.37 D
K2	42.86 D ± 2.77 D

**Table 5:** The type of astigmatism in children and adults.

	Children	Adults
Astigmatism type	80% WTR	82% WTR
Astigmatism type	20% OBL	11.8% OBL

**Table 6:** Axial length (AL) for gender.

					95% Confidence Interval				
	No of Eyes	Mean	SD	SE	Lower Bound	Upper Bound	Min	Max	*p* Value
Males	16	24.13	2.06	0.51	23.03	25.23	20.96	27.30	0.440 *
Females	16	23.46	1.05	0.26	22.90	24.02	21.20	24.80	
Total	32	23.79	1.64	0.29	23.20	24.39	20.96	27.30	

* Wilcoxon test between males and females.

**Table 7:** Axial length (AL) for age.

	Children	Adults	All Cases	*p* Value
AL < 22 mm	20%	11.8%	15.6%	0.213 *
AL 22–25 mm	66.7%	76.5%	71.9%	
AL > 25 mm	13.3%	11.8%	12.5%	

* Mann–Whitney test between children and adults.

## Data Availability

Data are available on request from the corresponding author.
